# Phytochemical and Pharmacological Profiles of Three *Fagopyrum* Buckwheats

**DOI:** 10.3390/ijms17040589

**Published:** 2016-04-19

**Authors:** Rui Jing, Hua-Qiang Li, Chang-Ling Hu, Yi-Ping Jiang, Lu-Ping Qin, Cheng-Jian Zheng

**Affiliations:** 1 Department of Pharmacognosy, School of Pharmacy, Second Military Medical University, Shanghai 200433, China; jingruisoda@163.com (R.J.); lihuaqiang2009@126.com (H.-Q.L.); msjyp@163.com (Y.-P.J.); qinsmmu@126.com (L.-P.Q.); 2 Department of Natural Products Chemistry, School of Pharmacy, Fudan University, Shanghai 201203, China; changfeih@126.com

**Keywords:** *Fagopyrum*, buckwheat, phytochemistry, pharmacology

## Abstract

The genus *Fagopyrum* (Polygonaceae), currently comprising 15 species of plants, includes three important buckwheat species: *Fagopyrum esculentum* (*F. esculentum*) Moench. (common buckwheat), *Fagopyrum tataricum* (*F. tataricum*) (L.) Gaertn. (tartary buckwheat) and *Fagopyrum dibotrys* (*F. dibotrys*) (D. Don) Hara. (perennial buckwheat), which have been well explored due to their long tradition of both edible and medicinal use. This review aimed to present an up-to-date and comprehensive analysis of the phytochemistry and pharmacology of the three *Fagopyrum* buckwheats. In addition, the scope for future research was also discussed. All available references included in this paper were compiled from major databases, such as MEDLINE, Pubmed, Scholar, Elsevier, Springer, Wiley and CNKI. A total of 106 compounds isolated from three *Fagopyrum* buckwheats can be mainly divided into six classes: flavonoids, phenolics, fagopyritols, triterpenoids, steroids and fatty acids. Flavonoids and phenolic compounds were considered to be the major active components. Considerable pharmacological experiments both *in vitro* and *in vivo* have validated that *Fagopyrum* buckwheats possess antitumor, anti-oxidant, anti-inflammatory, hepatoprotective, anti-diabetic activities, *etc.* All reported data lead us to conclude that *Fagopyrum* buckwheats have convincing medicinal potential. However, further research is needed to explore its bioactive constituents, the relationship to their structural activities and the molecular mechanisms of action.

## 1. Introduction

The genus *Fagopyrum,* a member of family Polygonaceae, comprises 15 species that are mainly distributed in the North Temperate Zone. A total of 10 species and one variety occur in China, including three important buckwheat species: *Fagopyrum esculentum* (*F. esculentum*) Moench. (common buckwheat), *Fagopyrum tataricum* (*F. tataricum*) (L.) Gaertn. (tartary buckwheat), and *Fagopyrum dibotrys* (*F. dibotrys*) (D. Don) Hara. (perennial buckwheat) [1]. Due to their long tradition of both edible and versatile medicinal use, more and more chemical and pharmacological studies have been carried out on the above-mentioned three buckwheat species and little research has been performed on other *Fagopyrum* species.

*F. esculentum* is an annual Asian herb with clusters of small pinkish or white flowers and edible triangular seeds, while *F. tataricum* is also an erect annual herb but with smaller seed size. *F. esculentum* and *F. tataricum* are two important crop plants and their seeds are consumed as the main buckwheats worldwide, as a potential “functional food” material [2,3], particularly due to their high quality protein, abundant phenolic compounds and well balanced essential amino acids and minerals [4,5]. Detailed comparisons of the seeds of *F. esculentum* and *F. tataricum* revealed that the former has advantages of sweet taste, large seed size, and easy dehulling of seed coat. In contrast, the latter is of bitter taste and small size with tight seed coat. Despite the above-mentioned disadvantages, *F. tataricum* has been reported to contain much more phenolics content than *F. esculentum* [3,6,7,8], which is therefore being increasingly favored by researchers in recent years [9,10,11]. Besides their edibility, both *F. esculentum* and *F. tataricum* were also traditionally used in folk medicine for various medicinal purposes. According to Traditional Chinese Medicine (TCM) theory, *F. esculentum* seeds possess the ability of invigorating the spleen, eliminating food stagnating and descending *qi*-flowing. It has also been cited as an anti-hemorrhagic and hypotensive drug in the British Herbal Pharmacopoeia and used as therapeutics for anti-inflammation, detoxification and lowering the fever in Korean folk medicine [12,13], whereas *F. tataricum* seeds have been found in wide use for antioxidant, antitumor, hypoglycemic and hypolipidemic purposes [14,15,16,17,18]. In addition, *F. tataricum* roots were traditionally utilized to regulate *qi*-flowing for relieving pain, invigorate the spleen and drain dampness, which were commonly used to treat some chronic and incurable diseases, such as rheumatic disorders, cancers and general debility. Its roots were therefore called “*Qiao ye qi*”, indicating that it might have similar effects as “*San qi*” (*Panax notoginseng* roots) [10,14,19,20], which prompted our group to investigate the bioactive metabolites from *F. tataricum* roots and subsequently revealed the abundance of cytotoxic phenylpropanoid glycosides that have potential use in cancer therapy [9]. *F. dibotrys*, also called *F. cymosum* (Trev.) Meisn, is an erect perennial herb with edible seeds and leaves that are rich in rutin, which makes a healthy addition to the diet [21,22,23]. Its leaves can be boiled or steamed and used like spinach [24]. In China, its rhizome was regarded as folk medicine for clearing away heat and toxic materials, removing blood stasis and expelling pus, which was generally used for the treatment of lung diseases, rheumatism, cancer, dysmenorrhea, inflammation, lumbago, snakebite and traumatic injuries, especially effective for lung cancer [25,26,27].

To the best of our knowledge, modern pharmacological studies revealed that the above-mentioned three *Fagopyrum* species possessed versatile bioactivities, including anti-tumor, anti-oxidant, anti-inflammatory, anti-aging, hepatoprotective, hypoglycemic, anti-allergic, anti-fatigue activities, *etc.* [12,25,26,28,29,30,31]. Several types of bioactive phenolics including flavonoids, condensed tannins, phenylpropanoids and phenol derivatives were isolated from those buckwheat species. Flavonoids in *Fagopyrum* buckwheats exhibited remarkable antioxidant and cardio-cerebral vascular protective effects [32,33,34] and thus these buckwheats were considered as valuable dietary supplements. The condensed tannins, isolated from the rhizomes of *F. dibotrys*, showed excellent anti-tumor and anti-oxidant effects [33,35,36,37,38]. Additionally, phenylpropanoid glycosides were found to be the major bioactive constituents in *F. tataricum* roots, which displayed significant cytotoxicity [9].

Due to the prominent values of these three *Fagopyrum* species on both edible and medicinal uses, in this paper, we have documented an up-to-date and comprehensive retrospection of the phytochemical and pharmacological studies on these three *Fagopyrum* buckwheats, which provided a scientific basis for further studies on these species.

## 2. Phytochemicals

### 2.1. Flavonoids

**Figure 1:**
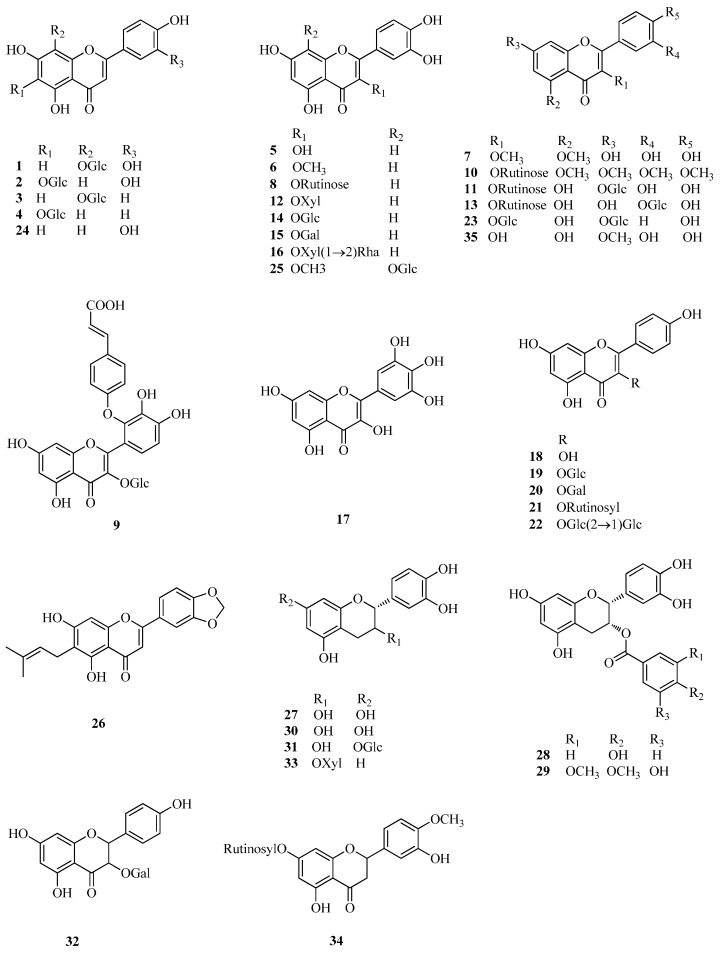
Structures of flavonoids isolated from *Fagopyrum esculentum* (*F. esculentum*), *Fagopyrum tataricum* (*F. tataricum*) and *Fagopyrum dibotrys* (*F. dibotrys*).

**Figure 2:**
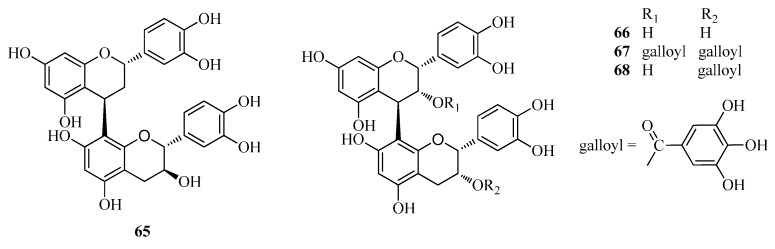
Structures of tannin compounds isolated from *F. esculentum*, *F. tataricum* and *F. diabotrys*.

Flavonoids, a group of polyphenolic compounds consisting of a 15-carbon basic skeleton (C6–C3–C6), found widely in plants and human diet, are potent antitumor, antioxidants and microcirculation improver [32]. Flavonoids have been proven to be the major active compounds in *Fagopyrum* buckwheats, and the class and content of flavonoids varied in different parts of *Fagopyrum* buckwheats. For example, six flavonoids (rutin (**8**), quercetin (**5**), orientin (**1**), vitexin (**3**), isovitexin (**4**) and isoorientin (**2**)) were found in *F. esculentum* hulls, while only rutin (**8**) and isovitexin (**4**) were found in the seeds. In addition, in all tested tissues, much more content of rutin was found in flowers than that in stems and leaves [39]. Since the discovery of rutin in *F. esculentum* in the 20th century, more than 30 flavonoids have been isolated and identified from these *Fagopyrum* buckwheats, such as aromadendrin-3-*O*-d-galactoside (**32**) and taxifolin-3-*O*-d-xyloside (**33**) from *F. esculentum* [40] 3-methyl-gossypetin-8-*O*-β-d-glucopyranoside (**25**) and quercetin-3-*O*-(2′′-*O*-*p*-hydroxy-coumaroyl)-glucoside (**9**) from *F. dibotrys* [33] 5,7,3′,4′-tetramethylquercetine-3-*O*-rutinoside (**10**) and quercetine-3-*O*-rutinoside-7-*O*-galactoside (**11**) from *F. tataricum* [41] and so on, most of which were obtained as *O*-glycosides (Figure 1). In addition, catechins (flavanols) and condensed tannins (proanthocyanidins) were also found in these *Fagopyrum* buckwheats. (−)-Epicatechin (**27**), (−)-epicatechin-3-*O*-*p*-hydroxybenzoate (**28**), (−)-epicatechin-3-*O*-(3,4-di-*O*-methyl)-gallate (**29**), and (+)-catechin-7-*O*-glucoside (**31**) were found in *F. esculentum* [34] while (+)-catechin (**30**) and (−)-epicatechin (**27**) were reported from *F. dibotrys* [33]. Four major condensed tannins, dimers of catechin derivatives, including procyanidin B-1 (**65**), procyanidin B-2 (**66**), 3,3-di-*O*-galloyl-procyanidin B-2 (**67**), and 3-*O*-galloyl-procyanidin B-2 (**68**), were isolated from the rhizomes of *F. dibotrys* and displayed significant radical-scavenging activities. Especially, 3,3-di-*O*-galloyl-procyanidin B-2 (**67**) and 3-*O*-galloyl-procyanidin B-2 (**68**) were the most active ones due to their abundance of phenolic hydroxyl groups [33]. The chemical names and plant sources of these compounds (**1**–**35**) are shown in Table 1 and Figure 1 and Figure 2.

### 2.2. Phenolics

**Figure 3:**
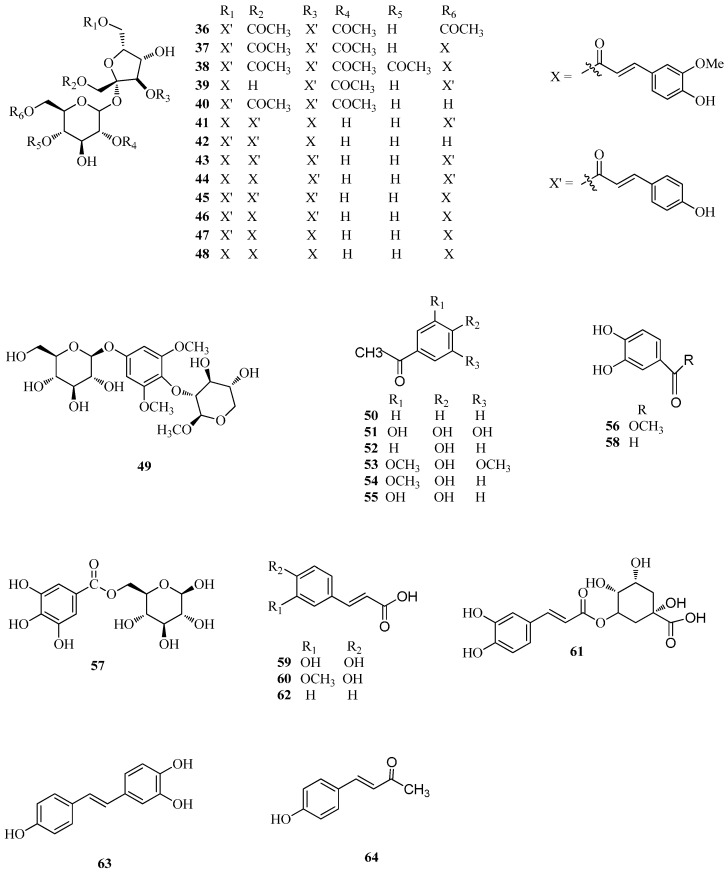
Structures of phenolic compounds isolated from *F. esculentum*, *F. tataricum* and *F. diabotrys*.

Phenolic compounds are secondary metabolites derivated from the pentose phosphate, shikimate, and phenylpropanoid pathways in plants [59]. This class of compounds exhibits a wide range of physiological properties (antioxidant, antitumor, antibacterial activities, *etc.*), and is ubiquitous in plants [60]. The major phenolic constituents in these *Fagopyrum* buckwheats include phenylpropanoids and derivatives of hydroxybenzoic and hydroxycinnamic acid. Our group has recently isolated and identified seven new phenylpropanoid glycosides, tatarisides A–G (**36**–**42**), with potent cytotoxic activity, from the roots of *F. tataricum* [9] together with diboside A (**43**), a phenylpropanoid previously isolated from the rhizomes of *F. dibotrys* along with lapathoside A (**44**) possessing the same skeleton. More recently, 1,3,6,6′-tetra-feruloyl sucrose (**48**), a new phenylpropanoid glycoside named taroside, was isolated from *F. tataricum* seeds together with 3,6-di-*p*-coumaroyl-1,6′-di-feruloyl sucrose (**46**), 1,3,6′-tri-feruloyl-6-*p*-coumaroyl sucrose (**47**), and 1,3,6-tri-*p*-coumaroyl-6′-feruloyl sucrose (**45**) [45]. More detailed information on phenolic compounds is listed in Table 1 and shown in Figure 3.

### 2.3. Fagopyritol

**Figure 4:**
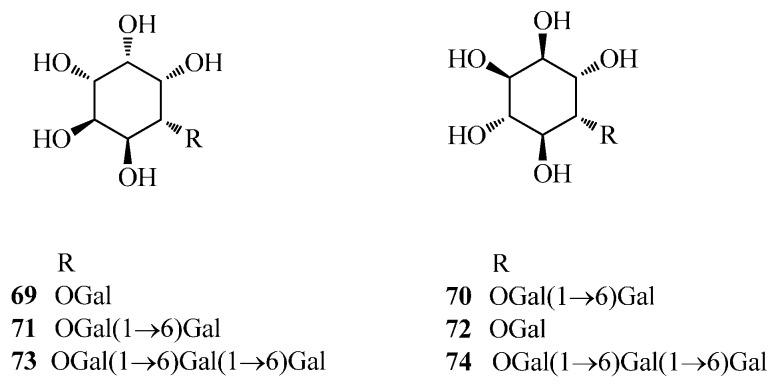
Structures of cyclitol compounds isolated from *F. esculentum*, *F. tataricum* and *F. diabotrys*.

Fagopyritols are mono-, di-, and trigalactosyl derivatives of D-*chiro*-inositol that are accumulated in embryo and aleurone tissues of buckwheat seeds and may be important for seed maturation and as a dietary supplement. Thus far, a total of six fagopyritols (fagopyritols A1, A2, A3, B1, B2 and B3) have been identified from *F. esculentum* seeds [53,54], classified into two series of fagopyritol oligomers based on the linkage between galactopyranosyl and D-*chiro*-inositol moiety. Fagopyritols A1 (**69**), A2 (**70**) and A3 (**71**) were of A-series with a 1→3 linkage, identified as α-d-gal-(1→3)-d-*chiro*-inositol (**69**), α-d-gal-(1→6)-α-d-gal-(1→3)-d-*chiro*-inositol (**70**), and α-d-gal-(1→6)-α-d-gal-(1→6)-α-d-gal-(1→3)-d-*chiro*-inositol (**71**), respectively, while fagopyritols B1 (**72**), B2 (**73**) and B3 (**74**), with a 1→2 linkage, were identified as α-d-gal-(1→2)-d-*chiro*-inositol (**72**), α-d-gal-(1→6)-α-d-gal-(1→2)-d-*chiro*-inositol (**73**), and α-d-gal-(1→6)-α-d-gal-(1→6)-α-d-gal-(1→2)-d-*chiro*-inositol (**74**), respectively. Fagopyritol A1 (**69**) and fagopyritol B1 (**72**) are the prominent fagopyritols accumulated and can facilitate desiccation tolerance and storability of buckwheat seeds [54,61]. Moreover, fagopyritols are structurally similar to a galactosamine derivative of d-*chiro*-inositol, a putative insulin mediator [62] and therefore may be useful in the treatment for non-insulin dependent diabetes mellitus [61]. More detailed information on Fagopyritols (**69**–7**4**) is listed in Table 1 and shown in Figure 4.

### 2.4. Triterpenoids

**Figure 5:**
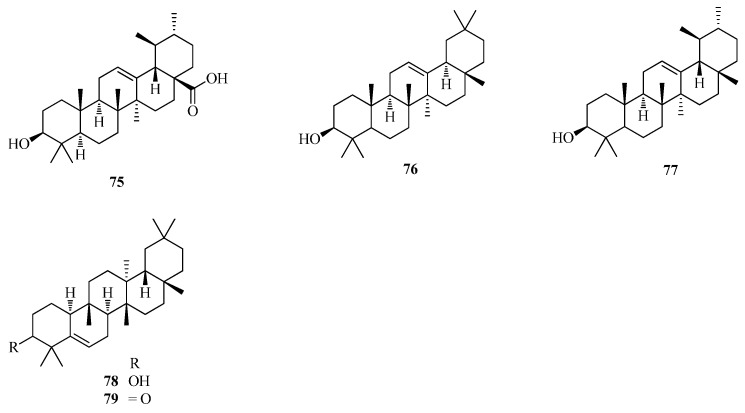
Structures of triterpenoids isolated from *F. esculentum*, *F. tataricum* and *F. diabotrys*.

A few triterpenoids (**75**–**79**) have been reported from *Fagopyrum* buckwheats. Glutinone and glutinol were isolated from the rhizomes of *F. dibotrys* [48] while olean-12-en-3-ol (**76**) and urs-12-an-3-ol (**77**) were identified from *F. esculentum* seed oil that was extracted with petroleum ether and analyzed by capillary GC/MS [56]. In addition, ursolic acid (**75**) was isolated from *F. dibotrys* [33]. The names and structures of triterpeniods are listed in Table 1 and shown in Figure 5.

### 2.5. Steroids

**Figure 6:**
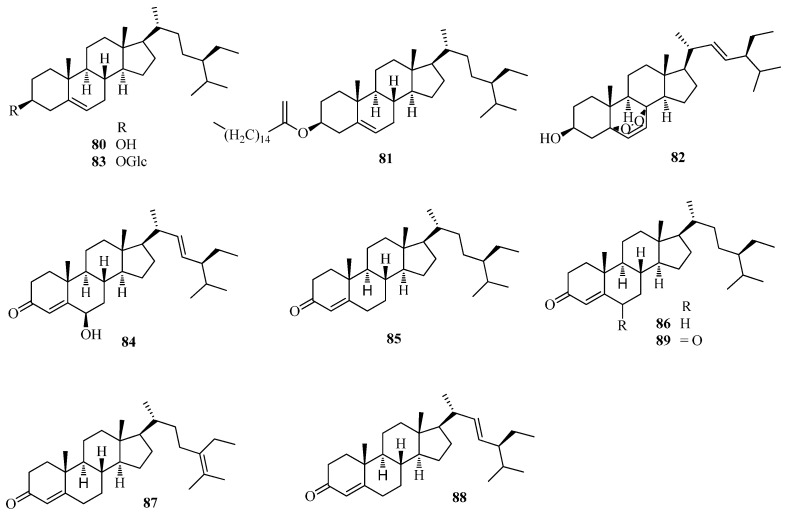
Structures of steroids isolated from *F. esculentum*, *F. tataricum* and *F. diabotrys*.

A total of five steroids, including β-sitosterol (**80**), β-sitosterol palmitate (**81**), ergosterol peroxide (**82**), daucosterol (**83**) and stigmsat-4-en-3,6-dione (**89**), were isolated from the seeds of *F. tataricum* [49]. Other steroids were identified as 6-hydroxystigmasta-4,22-dien-3-one (**84**), 23*S*-methylcholesterol (**85**), stigmast-5-en-3-ol (**86**), stigmast-5,24-dien-3-ol (**87**), and *trans*-stigmast-5,22-dien-3-ol (**87**) in *F. esculentum* seed oil by capillary GC/MS [56]. Hecogenin was isolated from *F. dibotrys* [63]. All steroid compounds are listed in Table 1 and shown in Figure 6.

### 2.6. Fatty Acid

**Figure 7:**
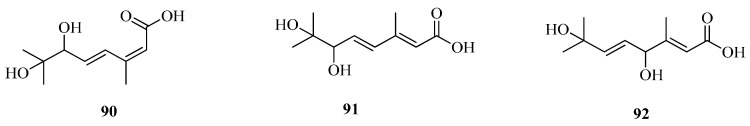
Structures of fatty acids isolated from *F. esculentum*, *F. tataricum* and *F. diabotrys*.

This class of compounds is of minor polarity in plants. Fifteen fatty acids were determined in *F. esculentum* seed oil analyzed by capillary GC/MS [56]. In addition, three new fatty acids, 6,7-dihydroxy-3,7-dimethyl-octa-2(*Z*),4(*E*)-dienoic acid (**90**), 6,7-dihydroxy-3,7-dimethyl-octa-2(*E*),4(*E*)-dienoic acid (**91**) and 4,7-dihydroxy-3,7-dimethyl-octa-2(*E*),5(*E*)-dienoic acid (**92**) were isolated from the methanol extract of *F. esculentum* hulls. These compounds, at 500 µg/mL, showed potential antimicrobial activity against *Staphylococcus aureus* [57]. The names and structures of three compounds are listed in Table 1 and shown in Figure 7.

### 2.7. Volatile Compounds

Buckwheats have a strong characteristic aroma. Volatile constituents are believed to play a major role in the buckwheat aroma. Volatiles from ground *F. esculentum* flour were analyzed by GC/MS, among which, 2,5-dimethyl-4-hydroxy-3(2H)-furanone, (*E*,*E*)-2,4-decadienal, phenylacetaldehyde, 2-methoxy-4-vinylphenol, (*E*)-2-nonenal, decanal, hexanal and salicylaldehyde were regarded as the major contributors to the buckwheat aroma compounds [64]. In addition, the aroma-active components of commercially obtained “monofloral” buckwheat honey, at least 51% the constituent nectar or 45% of contaminant pollen were from a single floral source (*F. esculentum*), were identified by Gas chromatography-olfactometry (GCO) of decreasing headspace samples, which revealed that the most aroma-active odorants were 3-methylbutanal, 3-hydroxy-4,5-dimethyl-2(5H)-furanone (sotolon) and (*E*)-β-damascenone, with 3-methylbutanal being primarily responsible for the distinct malty aroma [65].

### 2.8. Other Compounds

**Figure 8:**
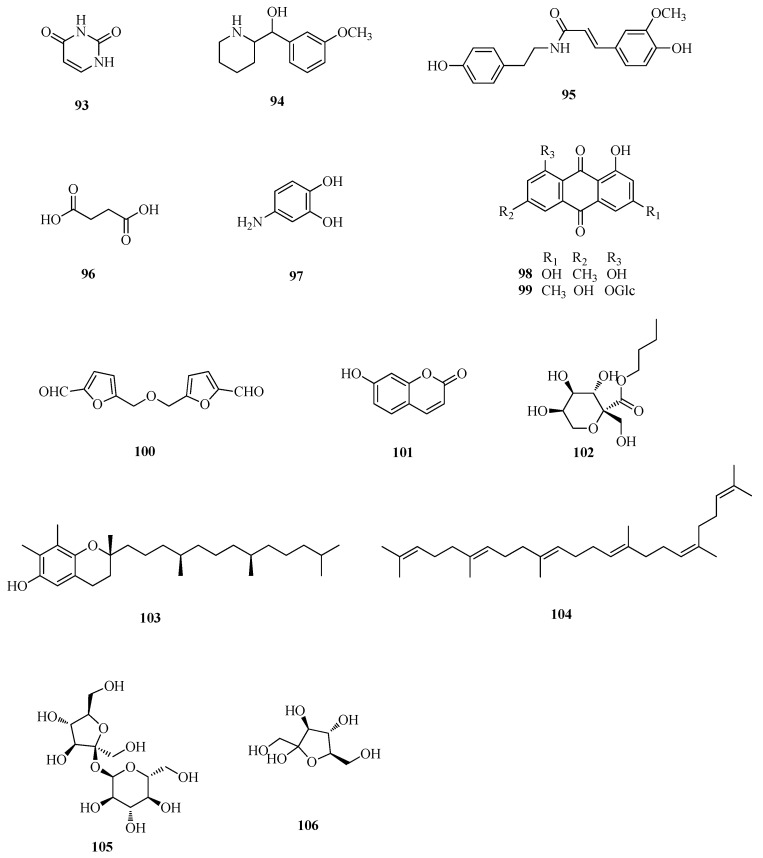
Structures of other compounds isolated from *F. esculentum*, *F. tataricum* and *F. diabotrys*.

Alkaloids, anthraquinones, coumarins and carbohydrate derivatives were also reported from these three buckwheats. Uracil (**93**), 3,4-dihydroxy benzamine (**97**), 5,5′-di-α-furaldehyde dimethyl ester (**100**), sucrose (**105**) and fructose (**106**) were isolated from seeds of *F. tataricum*. (3-Methoxyphenyl)-2-piperidinemethanol (**94**), *n*-butyl-β-d-fructopyranoside (**95**), γ-tocopherol (**103**) and squalene (**104**) were isolated from *F. esculentum*. Succinic acid (**96**), emodin (**98**), emodin-8-*O*-β-d-glucopyranoside (**99**), and 7-hydroxycoumarin (**101**) were isolated from the rhizomes of *F*. *dibotrys* [33,42,46,49,51]. In addition, these three buckwheats also possess abundant nutritional protein with well-balanced essential amino acids (glutamic acid, arginine, asparaginic acid, glycine, lysine, *etc*.) and minerals (K, Mg, Na, Zn, Ca, Mn, *etc*.) [27]. More detailed information is listed in Table 1 and shown in Figure 8.

## 3. Pharmacological Properties

These three *Fagopyrum* buckwheats possessed versatile bioactivities, reputed for their anti-tumor [9,12,14,26,63,66,67,68,69,70,71,72,73,74,75,76,77,78,79,80,81,82,83,84], anti-oxidant [6,28,38,40,85,86,87,88,89,90,91,92,93], anti-inflammatory [94,95,96,97], hepatoprotective [29,98,99,100,101,102,103], anti-hyperglycemic [30,104,105,106,107,108,109,110,111,112], anti-allergic [12], anti-bacterial [42,108,109,110,111,112,113,114,115] and anti-fatigue activities [116,117]. In this section, the available pharmacological data on *F. esculentum*, *F. tataricum*, and *F. dibotrys* have been documented.

### 3.1. Antitumor Activity

*F. dibotrys* (*F. cymosum*) has been used in China to treat various lung ailments for a long time, including lung tumors. *In vitro* experiments revealed that a commercial extract of *F. Cymosum* obtained from the International Herbal Pharmaceuticals Inc. (Whitestone, New York, NY, USA), exhibited broad-spectrum cytotoxicity, significantly inhibiting the growth of cancer cells from lung (H460), liver (HepG2), colon (HCT116), leukocytes (K562) and bone (U2OS) with concentrations that cause 50% inhibition of cell growth (*G_50_*) approximately in the range of 25–40 μg/mL, whereas cancer cells derived from prostate (DU145), cervix (HeLa-S3), ovary (OVCAR-3) and brain (T98G) were not sensitive to *F. cymosum*. Synergistic inhibition effect of *F. cymosum* and daunomycin was also observed in human lung cancer cells (H460) [25,26]. Many *in vivo* and *in vitro* studies revealed that Fr4 (the forth fraction from the extract of *F. cymosum* rhizomes) was responsible for the antiproliferative activity of *F. cymosum* and regarded as the most promising fraction. Fr4, with more than 50% phenolic content, significantly inhibited the growth of Lewis lung tumor on C57BL/6 mouse at a dose of 400 mg/kg, which down-regulated the expression of matrix metalloprotease (MMP-9) in mice [69]. Furthermore, Fr4 could suppress the growth of transplanted tumors of Sarcoma-180 (S180) and Hepatoma-22 (H22) in mice [66], which exhibited synergistic effect with cyclophosphamide and also alleviated the myelosuppression adverse effect of cyclophosphamide [67,68]. Dimer of 5,7,3’,4’-tetrahydroxyflavan-3-ol (C4–C8 linked), named dimeric procyanidin, was recognized as the major bioactive constituent of *F. cymosum* [63]. In addition, Fr4 induced HL-60 apoptosis through the down regulation of telomerase activity [72].

The combination of extracts of *F. cymosum* and *Rosa roxburghii* showed an *in vitro* synergistic effect on inhibiting the growth and inducing apoptosis of the human gastric carcinoma SGC-7901, pulmonary carcinoma A549 [75] and esophageal carcinoma CaEs-17 [79] cell lines. Co-administration of *F. cymosum* extracts and matrine could inhibit the expression of adhesion molecules (CD44, CD49, ICAM-1, and E-selectin), thus reducing the adhesion ability of high metastasis lung cancer cell line (PG) to human umbilical vein endothelial cell (HUVEC), and preventing tumor cells to adhere to vessel wall, which could also inhibit the invasion of human hepatocellar live carcinoma cell line (HepG2) by means of up-regulating the mRNA expression of nm23-H1 and down-regulating the expression of Tiam-1 [74].

Extract of *F. esculentum* flowers and leaves (EBFL) significantly inhibited tumor cell proliferation and induced the apoptosis of H22 cells [83,84]. Furthermore, it could alleviate cyclophosphamide (CTX)-induced immunosupression by boosting the immune function of H22 tumor mice [82]. EBFL could also inhibit the growth of S180 tumor in mice, which may be related to the increasing GSH-Px and SOD activity and decreased malondial dehyde (MDA) content [80]. *In vitro*, it also inhibited the HL-60 cell growth and blocked cells from G_0_/G_1_ to S phase [83]. The ethyl acetate and butanol fraction of *F. esculentum* sprout ethanol extract, at the concentration of 1.0 mg/mL, also showed strong cytotoxicity against A549, AGS, MCF-7, Hep3B and Colo205 cancer cell lines with growth inhibition of 70.3%, 94.8%, 79.6%, 82.3%, and 73.2%, respectively [81]. In addition, recombinant buckwheat trypsin inhibitor (rBTI) possessed potent antiproliferative activity *in vitro* and its mutant (aBTI) displayed much stronger antiproliferative efficacy *in vitro* against HL-60, EC9706 and HepG2 cells and might be a novel candidate for cancer treatment [77,78]. A population-based case-control study revealed that intake of buckwheat was associated with reduced risk of lung cancer [73].

A novel cytotoxic protein, coded as TBWSP31, was isolated from the water-soluble extract of *F. tataricum*. TBWSP31 significantly inhibited the growth of human mammary cancer cell Bcap37 in time and concentration dependent manner, with *IC_50_* values of 43.37 (48 h) mg/mL and 19.75 mg/mL (72 h), via induction of apoptosis, up-regulation of Fas expression and down-regulation of B cell lymphoma 2 (Bcl-2) expression [10,14]. In addition, tartary buckwheat protein product (BWP) exhibited *in vivo* antitumor activity against 1,2-dimethylhydrazine (DMH)-induced colon carcinogenesis by suppressing cell proliferation [71] and also retarded 7,12-dimethylbenz[α]anthracene-induced mammary carcinogenesis in rat [70]. In addition, our recent work revealed that seven new phenylpropanoid glycosides, tatarisides A–G (**36**–**42**), from the roots of *F. tataricum*, exhibited potent cytotoxic activity against four human cancer cell lines (A-549, HCT116, ZR-75-30 and HL-60) with *IC_50_* values in the range of 2.83–55.66 μg/mL [9].

### 3.2. Anti-Oxidant Activity

Many phenolic compounds, including flavonoids, tannins, phenolic acids, coumarins, lignans, stilbenes, and curcuminoids, have been reported to possess potent antioxidant activity [86]. The wide use of *Fagopyrum* buckwheats as medicinal food largely benefits from the abundance of phenolic compounds [38]. Rutin (**8**) was early found to be rich in *F. esculentum* and showed significant antioxidant activity. At the concentration of 0.05 mg/mL, ascorbic acid (Vc), butylated hydroxytoluene (BHT) and rutin (**8**) exhibited 92.8%, 58.8%, and 90.4% inhibition against 1,1-diphenyl-2-picryl-hydrazyl (DPPH) radical, respectively, and rutin (**8**) also showed effective inhibition on lipid peroxidation [89].

The extracting solvents of different polarities significantly affected the yield, total phenolics and antioxidant activity of buckwheat (*F. esculentum*) extracts. For example, the methanolic extracts showed the highest antioxidant activity coefficient (AAC) of 627.0 ± 40.0 at 200 mg/L by the β-carotene bleaching method and the longest induction time of 7.0 ± 0.2 h by the Rancimat method, whereas the acetone extract showed highest scavenging activity of 78.6 ± 6.2% at 0.1 mg/mL by the DPPH method [28]. The content of both rutin and total flavonoids significantly varied depending on species, 0.02% and 0.04% in *F. esculentum*, 0.10% and 0.35% in *F. homotropicum*, and 1.67% and 2.04% in *F. tataricum*, respectively. The results showed that the antioxidant activity decreased in the order: *F. tataricum* > *F. homotropicum* > *F. esculentum*. According to this, the contents of rutin and total flavonoids in buckwheats play an important role in antioxidant activity [6]. The phenolic content and antioxidant activity of tartary buckwheat also varied from different locations, revealing that growing conditions and the interaction between variety and environment contributes importantly to individual phenolics and antioxidant properties of tartary buckwheat [92]. However, different processing, such as roasting or extrusion, did not cause obvious change in total phenolic content and antioxidant activity of buckwheat flour. Roasted (200 °C, 10 min) buckwheat flour only exhibited an increase both in non-polar compounds and polar compounds, whereas extrusion exhibited increase only in polar compounds [87]. Besides buckwheat seeds, other parts of buckwheat also displayed significant antioxidant activity. The ethanolic extract of buckwheat hulls inhibited lipid peroxidation and five antioxidant compounds were isolated from its bioactive fractions, which were identified as quercetin (**5**), hyperin (**14**), rutin (**8**), protocatechuic acid (**55**), and 3,4-dihydroxybenzaldehyde (**5**) [40]. The hulls, bran and protein hydrolysates of *F. esculentum* exhibited an excellent antioxidant effect, including free radical scavenging ability and linoleic acid peroxidation inhibiting ability [85,90,91,93].

In addition, due to their significant antioxidant activities, both *F. esculentum* and *F. tataricum* ethanolic extracts remarkably inhibited the non-site-specific hydroxyl radical-mediated DNA damage and site-specific hydroxyl radical-mediated DNA strand breaks *in vitro*. The ethanolic extract of *F. tataricum* possessed higher content of phenolics and therefore exhibited stronger antioxidant activity than that of *F. esculentum* [88].

### 3.3. Anti-Inflammatory Activity

The ethanol extract of *F. esculentum* sprouts (ExtBS) showed significant anti-inflammatory activity both *in vitro* and *in vivo*. ExtBS can down-regulation IL-6 and TNF-α level in mice stimulated by LPS. Besides, it directly affected the gene expression of IL-6 and IL-8 in HeLa cells. In a word, ExtBS can be a promising candidate used to prevent the progress of various inflammatory diseases [94]. The 80% ethanol extract of *F. dibotrys* roots was suspended in water and partitioned with petroleum ether (PE), CHCl_3_, EtOAc, and n-BuOH, successively, which were subsequently screened for anti-inflammatory activity. The results indicated that the CHCl_3_ fraction was the most effective and significantly inhibited the auricle swelling in mice, voix pedis engorgement in rat and decrease the PGE2 level in rat swelling foot [96]. The 50% ethanol extract of *F. dibotrys* roots, with (−)-epicatechin (**27**) as the main bioactive constituent (2.18 mg/g), can also significantly restrain the mouse ear swelling induced by dimethylbenzene compared with model control group [95]. In addition, the extract of *F. cymosum* roots (Fag) significantly inhibited the acetic acid induced writhing in mice, and reduced the peritoneal permeability and the exudation of Evans blue in mice, indicating that Fag possessed antinociceptive and anti-inflammatory effects [97].

### 3.4. Heptoprotective Activity

The ethanol extract of germinated seeds of *F. esculentum*, with rutin (**8**) content increased more than 10 times and production of quercitrin (**12**) and one newly formed flavonoid after 48 h germination, displayed potent anti-fatty liver activities, which significantly reduced the triglyceride (TG) and total cholesterol (TC) levels in the liver of mice with a high-fat diet, by suppressing expression of the key adipogenic transcriptional factors, such as PPARγ and C/EBPα in hepatocytes [29].

The 60% ethanol extract of *F. tartaricum* seeds was shown to strikingly lower the activities of serum aminotransferase (ALT) and aspartate aminotransferase (AST) in a dose dependent manner in mice with acute liver injury induced by carbon tetrachloride (CCl_4_) and d-galactosamine. Significant alleviation of the histopatholocal changes in the liver was also observed in mice pretreated with *F. tartaricum* seeds extract before the induction of liver injury [101,102]. The 75% ethanol extracts from tartary buckwheat (EEB) also showed significant heptoprotective activity against ethanol- and CCl_4_-induced liver damage in C57BL/6 mice (ethanol induction) and Sprague-Dawley (SD) rats (CCl_4_ induction). EEB decreased the serum AST, ALT and alkaline phosphatase (ALP) levels in liver injured animals and enhanced the activities of antioxidant enzyme, including catalase (CAT), glutathione peroxidase (GPx), glutathione reductase (GR), and superoxide dismutase (SOD), and inhibited the levels of hepatic inflammation. All these suggested that EEB prevent hepatic injury via anti-oxidative and anti-inflammatory properties against oxidative liver damage. Rutin (**8**) and quercetin (**12**) displayed the similar effects as EEB and were considered as the major active compounds responsible for EEB’s heptoprotective activity [103]. In addition, *F. tartaricum* sprout powder exhibited a serum cholesterol-lowering effect by enhancing fecal bile acid excretion through increased fecal matter excretion or the up-regulation of hepatic cholesterol 7α-hydroxylase mRNA expression in rats [101,102]. Tartaricum protein extract exhibited significant hypocholesterolemic effect [98,99] and could suppressed gallstone formation *in vivo* [100].

### 3.5. Anti-Diabetic Activity

Tartary buckwheat (*F. tataricum*) was traditionally used to treat diabetes in China. A clinical observation revealed that intake of tartary buckwheat obvious alleviated the symptoms of both type I and type II diabetic patients, decreasing the levels of fasting blood glucose (FBG), glycosylated hemoglobin (GHb) and glycosylated serum protein (GSP), and increasing the level of fasting serum insulin [104]. d-chiro-inositol may be responsible for the antidiabetic activity of *F. tataricum*, which markedly ameliorated FBG of both diabetic Institute of Cancer Research (ICR) mice and patients [107]. The total flavonoid of tartary buckwheat could also lower the blood glucose, and increase the GSH level and Na-K-ATPase activity, and elevate the nerve conduction velocity and the blood flow in sciatic nerve after oral administration in streptozotocin induced diabetic rats, which indicated that tartary buckwheat flavonoids possessed favorable neuro-protective effects in diabetic rats [108,118]. In addition, complex *F. tataricum* prescription (CFTP), a traditional Chinese prescription for diabetes mellitus, significantly improved the symptoms of non-insulin-dependent diabetes mellitus (NIDDM) induced by streptozotocin injection in rats, with blood glucose, blood lipid and MDA decreased, SOD activities increased, and nitric oxide (NO) metabolism improved [105].

An epidemiological study revealed that intake of buckwheat (*F. esculentum*) in diet can lower the blood glucose concentration (BGC) and the prevalence rate of diabetes mellitus [106]. Both the ethanol and water extracts of *F. esculentum* seeds significantly reduced the blood glucose of normal and type II diabetes rats [109]. *F. esculentum* flowers and leaves also exhibited significant antidiabetic activity and rutin was considered as the main bioactive constituent, which could regulate the metabolic disorder of glucose and lipids in fat emulsion and alloxan-induced diabetic rats and improve insulin resistance [111] and possessed protective effect on liver injury at early stage in diabetic rats by decreasing the levels of FBG, serum TBil, ALT and liver index and restoring the histologica1 injury of hepatocytes [112]. In addition, buckwheat protein could lower the blood glucose in alloxan-induced diabetic mice [110], whereas co-asministration of pumpkin and buckwheat significantly reduced the blood glucose in alloxan-induced diabetic rats [119].

### 3.6. Antibacterial Activity

The EtOAc fraction from the ethanol extract of *F. dibotrys* significantly restrained the growth of beta *Hemolytic streptococcus* and *Pneumococcus* in petri dish dilution assay, which also exhibited favorable protection in mice infected with *Streptococcus pneumoniae*. Moreover, bioguided isolation of compounds from the EtOAc fraction yielded eight compounds, including *trans*-*p*-hydroxy cinnamic methyl ester (**64**), protocatechuic acid (**55**), protocatechuic acid methyl ester (**56**), 1uteolin (**24**), quercitrin (**12**), rutin (**8**), and (−)-epicatechin (**27**). These results indicate that phenolics and flavonoids were responsible for the bacteriostastic activity of *F. dibotrys* [42]. Recently, Dong *et al.* revealed that *F. dibotrys* possessed protective effect against lung injury induced by *Klebsiella pneumonia* in rats by down-regulation the mRNA expression of TLR2/4, MyD88 and MIP-2, and the protein expression of IκB-α, TNF-α, ICAM-1 and NFκB p65 in rat lung tissue [114,115]. In addition, a clinical report indicated that combination of *F. dibotrys* and ciprofloxacin exhibited better anti-pneumonia effect than ciprofloxacin used alone, due to the bacteriostastic, cough-relieving and expectorant activity of *F. dibotrys* [113].

### 3.7. Anti-Allergic Activity

Buckwheat (*F. esculentum*) grain extract (BGE), given orally, intraperitoneally or intradermally, significantly inhibited compound 48/80-induced vascular permeability evidenced by Evans blue extravasation. Oral administration of BGE displayed significant inhibition on passive cutaneous anaphylaxis stimulated by anti-dinitrophenyl IgE. BGE also possessed inhibitory potential on compound 48/80-induced histamine release from rat peritoneal mast cells *in vitro*. Furthermore, BGE suppressed the IL-4 and TNF-α mRNA induction by phorbol myristate acetate (PMA) and A23187 in human leukemia mast cells. All these results suggest that BGE exert anti-allergic action probably by inhibition of histamine release and cytokine gene expression in the mast cells [12].

### 3.8. Anti-Fatigue Activity

Tartary buckwheat (*F. tataricum*) protein consists of well-balanced amino acids with high biological values, such as hypocholesterolemic and antitumor activities. In addition, tartary buckwheat protein also possesses significant anti-fatigue ability and especially the globulin in buckwheat protein distinctly improved the swimming time, the climbing-pole time and the content of liver hepatin, which also reduced the content of blood lactic acid and urea. Furthermore, factor *F* analysis [116] revealed that the globulin in buckwheat protein had a low level of factor *F* due to its high content of branch chain amino acid (BCAA), thus inhibiting the formation of 5-hydroxytryptamine (5-HT) which can suppress the ability of movement [117].

### 3.9. Other Activities

Despite the above-mentioned activities, *Fagopyrum* buckwheats also disclaimed several other bioactivities. It has been found that *F. esculentum* polysaccharide could restrain the central nervous system, effectively inhibiting the spontaneous motion, reducing the latent period of falling asleep and prolonging the sleep time induced by sodium pentobarbital in mice [120]. *F. tataricum* flavones showed estrogen-like activity, which can be modified by *in vitro* simulated digestion [121]. The 50% ethanol extract of *F. dibotrys* rhizomes exhibited significant antitussive and expectorant activities, which can reduce the times of coughing induced by ammonia in mice, and also increase the secretion of phenol red in mice using tracheal phenol red test [95]. An acute toxicity test found that maximum tolerated dose in mice was approximately 8.0 g/kg, which was 166 times the human adult dosage, indicating high safety [95]. Additionally, *F. esculentum* buckwheat extract showed inhibitory effect on the progression of renal failure in nephrectomized rats by improving the state of oxidative stress and renal tissue lesions, and enhancing renal function [122].

## 4. Conclusions

The seeds of *F. esculentum* and *F. tataricum* are consumed widely in many countries and contain many beneficial ingredients for humans such as flavonoids and phenolics, commonly used to develop functional foods [2]. Recently, *F. tataricum* is much more popular because of its abundance and much higher content of phenolics than that of *F. esculentum* [3]. The rhizome of *F. dibotrys* was used as an antitumor and anti-inflammatory herb for a long time in China [25]. A commercial product, “Wei-Mai-Ning” (WMN) has already been developed from *F. dibotrys*, and displays significant activity [123]. These three *Fagopyrum* buckwheats have been well explored due to their long tradition of both edible and medicinal uses.

Herein, we documented the existing phytochemical and pharmacological studies on these three *Fagopyrum* buckwheats. Almost all their traditional uses have been validated by modern pharmacological studies, focusing on their anti-tumor, anti-oxidant, anti-inflammatory, hepatoprotective, anti-diabetic, antibacterial, anti-allergic, anti-fatigue activities, *etc*. Because of their versatile pharmacological properties, a large number of studies have been carried out on the chemical profile of these three *Fagopyrum* buckwheats. More than 100 compounds including flavonoids, phenolics, fagopyritols, triterpenoids, steroids and fatty acids have been isolated and identified. Flavonoids and phenolic compounds were considered to be the major active components and mainly responsible for most of their activities.

Due to the lack of clinical trials, there are few published data on clinical efficacy, toxicity or side effects of these buckwheats and their constituents. Comprehensive well-controlled and double-blind clinical trials are therefore urgently needed to validate the efficacy and safety. Better explanations of the mechanisms of action of different extracts and compounds, and an exhibition of the possible interactions between bioactive constituents and synthetic drugs are needed. Furthermore, the structure–activity relationship and the possible synergistic action among the bioactive compounds of this plant need to be fully elucidated before they are used in clinical practice. Additionally, limited studies have been carried out on the other *Fagopyrum* species. Because of the close relationship between *Fagopyrum* plants, they may produce similar secondary metabolites and thus possess similar therapeutic potentials. Thus, the other *Fagopyrum* species should also be thoroughly investigated so as to fully utilize the *Fagopyrum* medicinal resources.

## Figures and Tables

**Table 1:** Chemical constitutents from *Fagopyrum esculentum* (*F. esculentum*), *Fagopyrum tataricum* (*F. tataricum*) and *Fagopyrum dibotrys* (*F. dibotrys*).

No.	Compounds	Source	Reference
**Flavonoids**			
**1**	orientin	*Fe*, *Ft*	[3]
**2**	isoorientin	*Fe*, *Ft*	[3]
**3**	vitexin	*Fe*, *Ft*	[3]
**4**	isovitexin	*Fe*, *Ft*	[3]
**5**	quercetin	*Fd*, *Fe*, *Ft*	[33,40,42,43,44]
**6**	3-methylquercetin	*Fd*	[33]
**7**	3,5-dimethylquercetin	*Fd*	[33]
**8**	rutin	*Fd*, *Fe*, *Ft*	[33,40]
**9**	quercetin-3-*O*-(2′′-*O*-*p*-hydroxy-coumaroyl)-glucoside	*Fd*	[33]
**10**	5,7,3′,4′-tetramethylquercetine-3-*O*-rutinoside	*Ft*	[41]
**11**	quercetin-3-*O*-rutinoside-7-*O*-galactoside	*Ft*	[41]
**12**	quercitrin (quercetin-3-*O*-rhamnoside)	*Fd*, *Ft*	[33,45]
**13**	quercetin-3-*O*-rutinoside-3′-*O*-β-glucopyranoside	*Fd*, *Ft*	[46]
**14**	hyperin/isoquercitrin (quercetin-3-*O*-glucoside)	*Fe*	[40,45,47]
**15**	quercetin-3-*O*-β-d-galactoside	*Ft*, *Fe*	[40,45]
**16**	quercetin-3-*O*-[β-d-xyloxyl-(1→2)-α-l-rhamnoside]	*Ft*	[45]
**17**	myricetin	*Fe*	[47]
**18**	kaempferol	*Fd*, *Ft*	[43,44,48]
**19**	kaempferol-3-*O*-glucoside	*Ft*	[45]
**20**	kaempferol-3-*O*-galactoside	*Ft*	[45]
**21**	kaempferol-3-*O*-rutinoside	*Ft*	[42,44,49]
**22**	kaempferol-3-*O*-sophoroside	*Fe*	[40]
**23**	kaempferol-3-*O*-glucoside-7-*O*-glucoside	*Fe*	[40]
**24**	luteolin	*Fd*	[48]
**25**	3-methylgossypetin-8-*O*-β-d-glucopyranoside	*Fd*	[33]
**26**	3′,4′-methylenedioxy-7-hydroxy-6-isopentenyl flavone	*Fd*	[41]
**27**	(−)-epicatechin	*Fe*, *Fd*	[34]
**28**	(−)-epicatechin-3-*O*-*p*-hydroxybenzoate	*Fe*	[34]
**29**	(−)-epicatechin-3-*O*-(3,4-di-*O*-methyl)-gallate	*Fe*	[34]
**30**	(+)-catechin	*Fd*	[33]
**31**	(+)-catechin-7-*O*-glucoside	*Fe*	[34]
**32**	aromadendrin-3-*O*-d-galactoside	*Fe*	[40]
**33**	taxifolin-3-*O*-d-xyloside	*Fe*	[40]
**34**	hesperidin	*Fd*	[46]
**35**	rhamnetin	*Fd*	[46]
**Phenolics**			
**36**	tatariside A	*Ft*	[9]
**37**	tatariside B	*Ft*	[9]
**38**	tatariside C	*Ft*	[9]
**39**	tatariside D	*Ft*	[9]
**40**	tatariside E	*Ft*	[9]
**41**	tatariside F	*Ft*	[9]
**42**	tatariside G	*Ft*	[9]
**43**	diboside A	*Ft*, *Fd*	[33]
**44**	lapathoside A	*Fd*	[33]
**45**	1,3,6-tri-*p*-coumaroyl-6′-feruloyl sucrose	*Ft*	[45]
**46**	3,6-di-*p*-coumaroyl-1,6′-di-feruloyl sucrose	*Ft*	[45]
**47**	1,3,6′-tri-feruloyl-6-*p*-coumaroyl sucrose	*Ft*	[45]
**48**	taroside (1,3,6,6′-tetra-feruloyl sucrose)	*Ft*	[45]
**49**	1,3-dimethoxy-2-*O*-b-xylo-pyranosyl-5-*O*-β-glucopyranosyl-benzene	*Fd*	[50]
**50**	benzoic acid	*Fd*	[46]
**51**	gallic acid	*Fd*	[33]
**52**	*p*-hydroxybenzoic acid	*Fd*	[51]
**53**	syringic acid	*Ft*	[52]
**54**	vanillic acid	*Ft*	[52]
**55**	protocatechuic acid	*Fd*, *Fe*, *Ft*	[40,48]
**56**	protocatechuic acid methyl ester	*Fd*	[48]
**57**	6-*O*-galloyl-d-glucose	*Fd*	[33]
**58**	3,4-dihydroxybenzaldehyde	*Fd*, *Fe*	[40,48]
**59**	caffeic acid	*Ft*	[52]
**60**	ferulic acid	*Ft*	[52]
**61**	chlorogenic acid	*Fe*, *Ft*	[3]
**62**	*p*-coumaric acid	*Ft*	[52]
**63**	resveratrol	*Fe*	[40]
**64**	*trans*-*p*-hydroxy cinnamic methyl ester	*Fd*	[48]
**Tannins**			
**65**	procyanidin B-1	*Fd*	[33]
**66**	procyanidin B-2	*Fd*	[33]
**67**	3,3-di-*O*-galloyl-procyanidinB-2	*Fd*	[33]
**68**	3-*O*-galloyl-procyanidinB-2	*Fd*	[33]
**Cyclitol**			
**69**	fagopyritol A1	*Fe*	[53]
**70**	fagopyritol A2	*Fe*	[54]
**71**	fagopyritol A3	*Fe*	[54]
**72**	fagopyritol B1	*Fe*	[53]
**73**	fagopyritol B2	*Fe*	[55]
**74**	fagopyritol B3	*Fe*	[55]
**Triterpenoids**			
**75**	ursolic acid	*Ft*	[42,43]
**76**	olean-12-en-3-ol	*Fe*	[56]
**77**	urs-12-en-3-ol	*Fe*	[56]
**78**	glutinone	*Fd*	[48]
**79**	glutinol	*Fd*	[48]
**Steroids**			
**80**	β-sitosterol	*Ft*	[43,49]
**81**	β-sitosterol-palmitate	*Ft*	[49]
**82**	peroxidize-ergosterol	*Ft*	[49]
**83**	daucosterol	*Ft*	[43,49]
**84**	6-hydroxystigmasta-4,22-dien-3-one	*Fe*	[56]
**85**	23*S*-methylcholesterol	*Fe*	[56]
**86**	stigmast-5-en-3-ol	*Fe*	[56]
**87**	stigmast-5,24-dien-3-ol	*Fe*	[56]
**88**	*trans*-stigmast-5,22-dien-3-ol	*Fe*	[56]
**89**	stigmsat-4-en -3,6-dione	*Ft*	[49]
**Fatty Acids**			
**90**	6,7-dihydroxy-3,7-dimethyl-octa-2(*Z*),4(*E*)-dienoic acid	*Fe*	[57]
**91**	6,7-dihydroxy-3,7-dimethyl-octa-2(*E*),4(*E*)-dienoic acid	*Fe*	[57]
**92**	4,7-dihydroxy-3,7-dimethyl-octa-2(*E*),5(*E*)-dienoic acid	*Fe*	[57]
**Others**			
**93**	uracil	*Ft*	[49]
**94**	(3-methoxyphenyl)-2-piperidinemethanol	*Fd*	[48]
**95**	*N*-trans-feruloyltyramine	*Ft*	[45]
**96**	succinic acid	*Fd*	[51]
**97**	3, 4-dihydroxy benzamine	*Fd*	[48]
**98**	emodin	*Fd*	[49]
**99**	emodin-8-*O*-β-d-glucopyranoside	*Fd*	[33]
**100**	5, 5′-di-α-furaldehyde dimethyl ester	*Ft*	[51]
**101**	7-hydroxycoumarin	*Ft*	[11]
**102**	*n*-butyl-β-d-fructopyranoside	*Fd*	[48]
**103**	γ-tocopherol	*Fe*	[56]
**104**	squalene	*Fe*	[56]
**105**	sucrose	*Ft*	[58]
**106**	fructose	*Ft*	[58]

*Fe*, *Ft*, *Fd* are *Fagopyrum esculentum* Moench., *Fagopyrum tataricum* (L. Gaertn. (tartary buckwheat) and *Fagopyrum dibotrys* (D. Don) Hara., respectively.
